# Gut segments outweigh the diet in shaping the intestinal microbiota composition in grass carp *Ctenopharyngodon idellus*

**DOI:** 10.1186/s13568-019-0770-0

**Published:** 2019-04-06

**Authors:** Wenwen Feng, Jing Zhang, Ivan Jakovlić, Fan Xiong, Shangong Wu, Hong Zou, Wenxiang Li, Ming Li, Guitang Wang

**Affiliations:** 1 Key Laboratory of Aquaculture Disease Control, Ministry of Agriculture, and State Key Laboratory of Freshwater Ecology and Biotechnology, Institute of Hydrobiology, Chinese Academy of Sciences, Wuhan, 430072 China; 2 University of Chinese Academy of Sciences, Beijing, 100049 China; 3 Bio-Transduction Lab, Wuhan, 430072 China

**Keywords:** Gut microbiota, Diet, Midgut, Hindgut, Grass carp

## Abstract

Although dynamics of the complex microbial ecosystem populating the gastrointestinal tract of animals has profound and multifaceted impacts on host’s metabolism and health, it remains unclear whether it is the intrinsic or extrinsic factors that play a more dominant role in mediating the composition of intestinal microbiota. To address this, we studied the impacts of two strikingly different diets on a herbivorous fish, grass carp (*Ctenopharyngodon idellus*): a high-protein, low-fiber formula feed, and low-protein, high-fiber Sudan grass. After a 16-week feeding trial, microbial profiles of midgut and hindgut segments in both groups were compared. Bacterial composition was significantly different between the midguts of both groups, but not between the hindguts of two groups. Both PerMANOVA and VPA analyses suggested that gut segments explain a higher proportion of variation in intestinal microbiota than diet. Overall, our results suggest that intestinal compartments are a stronger determinant than diet in shaping the intestinal microbiota. Specifically, whereas diet has a strong impact on the composition of microbiota in proximal gut compartments, this impact is much less pronounced distally, which is likely to be a reflection of a limited ability of some microbial taxa to thrive in the anoxic environment in distal segments.

**Electronic supplementary material:**

The online version of this article (10.1186/s13568-019-0770-0) contains supplementary material, which is available to authorized users.

## Introduction

Gastrointestinal tract of animals harbors an extremely diverse and complex microbial ecosystem (Li et al. 2017; Torok et al. 2008; Wu et al. 2012; Xiong et al. 2017). In the course of coevolution of gut microbiota and hosts, gut microbial community has become an integral component of the host (Gilbert et al. 2012; Ley et al. 2008). Apart from contributing to the harvest of dietary nutrients that would otherwise be inaccessible to the host (Bäckhed et al. 2004; Rawls et al. 2006) and to the education of the host’s immune system (Miyake et al. 2015; Wu et al. 2011), they also have profound impacts on host’s development and behavior (Gacias et al. 2016).

Characterization of the intestinal microbiota and their ecological function is relatively advanced in humans and model mammals (Ley et al. 2006; Zhang et al. 2012), but less well understood in fish (Wu et al. 2012). Intestinal microbiota of fish are believed to be less complex and less numerous than those of terrestrial vertebrates (Miyake et al. 2015). Grass carp (*Ctenopharyngodon idellus*) is a globally distributed herbivorous fish (Feng et al. 2009) whose intestinal microbiota has been studied extensively in recent years (Han et al. 2010; Tran et al. 2017; Wu et al. 2012). *Proteobacteria*, *Firmicutes*, *Bacteroides*, *Actinobacteria*, and *Fusobacteria* are dominant in its intestine (Hao et al. 2017a; Ni et al. 2017; Tran et al. 2017). Recent investigations indicate that the intestinal microbiota of grass carp is likely to play an indispensable role in nutrient (especially polysaccharide) turnover and fermentation of the host (Hao et al. 2017b; Wu et al. 2015). Therefore, maintaining the homeostasis of intestinal microbiota is likely to be essential for health and survival of grass carp. The intestinal tract of grass carp is a simple coiled tube with eight convolutions, divided into three different segments according to its anatomical structure: foregut, midgut and hindgut (Ni and Wang 1999). Theoretically, physiological functions should be distinct in different intestinal regions: foregut is believed to be responsible for the absorption of lipids and hindgut for pinocytotic uptake of macromolecules, including proteins (Mowat and Agace 2014; Sire and Vernier 1992).

Although some members of microbiota are relatively constant (Faith et al. 2013), the overall composition of gut microbiota is very variable, and strongly influenced by extrinsic and intrinsic factors (Benson et al. 2010; Ley et al. 2008; Qin et al. 2010), resulting in notable variability among individuals (Wu et al. 2012). Regarding the extrinsic factors, diet is known to be a major determinant of the microbial community composition in both terrestrial (Ley et al. 2008) and aquatic (Carmody et al. 2015; Hao et al. 2017b; Ringø and Olsen 1999; Ringø et al. 2006; Tajima et al. 2001) vertebrates. Among the intrinsic factors (e.g. gut physiology, host’s phylogeny or genotype), gut segments are a strong predictor of the composition of intestinal microbial communities in terrestrial mammals (Ley et al. 2008; Perea et al. 2017; Zhang et al. 2018) and fish (Ye et al. 2014). However, relative contributions of diet and gut compartments have never been statistically tested in fish, so it still remains unclear whether it is the host’s gut segment or dietary intake that plays a more dominant role in mediating variations in the composition of intestinal microbiota.

To test these two hypotheses statistically, we set up a feeding trial using two very different diets: formula feed (high-protein, low-fiber) and Sudan grass (high-fiber, low-protein), and sampled microbial populations of midguts and hindguts of both diet groups after the feeding experiment. After the feeding trial, we compared microbial profiles of midgut and hindgut of both diet groups, and statistically tested the relative impacts of dietary intake and different gut segments on shaping the gut microbiota in the midgut and hindgut of grass carp. Therefore, the objectives of this work were two-fold: to infer differences in the microbial taxonomic composition among different intestinal compartments in grass carp, and to contribute to the understanding of relative contributions of diet and gut physiology on the microbial population structure in animals in general.

## Materials and methods

### Sample collection

Juvenile fish were purchased commercially and kept inartificial earthen ponds in Huanggang City, Hubei Province, China, from April to August, 2015. Six ponds (with 30 fish in each pond; 1.5–2.0 m depth, 100 m^2^ surface) were divided into two groups: one group was fed the Sudan grass diet (SG group) and the other was fed the formula feed diet (FF group). The Sudan grass diet contained 29% crude fiber and 10.37% crude protein, whereas the formula feed diet contained 6.9% crude fiber and 40.45% crude protein (Zhang et al. 2017). The fish were fed to apparent satiation twice a day (8:00 and 16:00 o’clock). After the feeding experiment (16 weeks), six grass carp specimens were randomly collected from each pond (6 × 6 = 36 specimens). Fishes were euthanized in buffered MS-222 at 250 mg/L concentration, measured (weight and length) and immediately dissected in sterile conditions. Body length was 30.67 ± 2.73 cm and weight was 486.57 ± 126.99 g. Intestines were divided into segments as described before (Ni and Wang 1999), the entire content of midgut and hindgut were collected, separately placed into labelled 25 mL polypropylene centrifuge tubes, frozen provisionally in a portable refrigerator, transported to laboratory within 6 h and stored at − 80 °C.

### DNA extraction, PCR amplification and sequencing

Genomic DNA was extracted from 72 samples (36 specimens × 2 gut segments) using QIAamp DNA stool mini kit (Qiagen, Germany Hilden) according to the manufacturer’s instructions. DNA concentrations were estimated using a Nanodrop 8000 Spectrophotometer (Thermo Scientific, Waltham, MA, USA). Obtained DNA samples were used for the amplification of bacterial V4-V5 16S rRNA gene region with universal barcode primers 515F (5′-GTGYCAGCMGCCGCGGTA-3′) and 909R (5′-CCCCGYCAATTCMTTTRAGT-3′) (Baker et al. 2003). PCR reaction mix (25 μL) contained 0.5 U of the Phusion high-fidelity DNA polymerase (New England Biolabs, Beijing China Ltd), 5 × Phusion GC buffer, 5 mM dNTP, 20 μM primers and 50 ng DNA. An initial denaturation at 98 °C for 30 s was followed by 25 cycles (98 °C for 10 s, 55 °C for 20 s and 72 °C for 20 s) and the final extension step for 10 min at 72 °C. PCR products were purified using AidQuick Gel Extraction Kit (Aidlab Biotech, Beijing, China). Purified samples were sequenced using Novogene bioinformatics technology on the Illumina Hiseq 2500 platform.

### Bioinformatic and statistical analyses

Raw sequenced data were analyzed using QIIME Pipeline-version 1.7.0 (Caporaso et al. 2010). Each sample was distinguished according to its unique barcode sequence (barcode mismatches = 0). The first processing step was merging paired-end reads using FLASH-1.2.8 program (Magoč and Salzberg 2011). Only the merged sequences with high-quality reads (length > 300 bp, without ambiguous base N, and average base quality score > 30) were used for further analyses. Sequence chimeras were removed using the UCHIME algorithm (Edgar et al. 2011). All sequences were grouped as operational taxonomic units (OTUs), applying a 97% identity threshold. Singletons and chloroplasts were filtered out. The sequence number of each sample was normalized to 11,000 sequences. All sequences analyzed in this study can be accessed in the SRA database under the accession number SRP 131857.

Samples (n = 72) were grouped using different criteria, diets (FF + SG, n = 36), gut segments (Midgut + Hindgut, n = 36), diet + segment (H-FF, M-FF, H-SG, M-SG; n = 18), and statistically analysed. Alpha diversity indices of gut bacterial communities, including community richness (Chao1 and Ace) and diversity (Shannon and Simpson), were calculated using the QIIME program. To evaluate the beta diversity and visualize differences in the bacterial community structure, principal coordinates analysis (PCoA) was conducted using the weighted UniFrac distance (Lozupone et al. 2011). To identify relative abundance of bacterial biomarker taxa at the genus level between the midgut and hindgut of different diet groups, linear discriminant analysis coupled with effect size (Lefse) was employed on the Huttenhower laboratory Galaxy website (http://huttenhower.sph.harvard.edu/galaxy/) (Segata et al. 2011). Default logarithmic (LDA) score value thresholds were set at 2.0 (to identify all significantly different taxa) and 4.0 (to generate publishable figures focusing only on the most significantly different taxa). Venn diagram was used to display shared OTUs between different parts of the intestine and different diets (Chow and Ruskey 2003). To reveal the similarities and differences among groups, a heatmap plot was constructed on the basis of the mean relative abundance of bacterial families which exceeded 0.1% in each sample. PICRUST 1.0 (Langille et al. 2013) and KEGG database were used to explore potential functional profiles of the bacteriome in different gut segments. Bar graph was constructed using OriginPro 8.5 (Stevenson 2015), and STAMP v2.1.3 (Parks et al. 2014) was used for statistical analyses of functional profiles. Statistical differences were calculated using Welch’s *t*-test with Bonferroni correction, with statistical significance threshold set at 0.05. Permutational multivariate analyses of variance (PerMANOVA) were performed using PAST 2.16 (Hammer-Muntz et al. 2001) to assess the significance of differences in the bacterial community structure among different groups, based on weighted UniFrac distance. PerMANOVA with ‘adonis’ procedure was used to evaluate whether the diet and the gut segment significantly affected the bacterial community structure of grass carp. Variance Partitioning Analysis (VPA) was used to evaluate the contribution of gut segments and diets to the microbial community variance.

All sequences analyzed in this study can be accessed in the SRA database under the accession number SRP 131857 (https://www.ncbi.nlm.nih.gov/Traces/study/?acc=SRP131857).

## Results

To test the hypothesis that diet should outweigh the intestinal segments in shaping the composition of microbial populations in grass carp, we set up a 16-week feeding experiment using two different diets: FF (high-protein, low-fiber) and SG (high-fiber, low-protein). Experimental setup comprised 18 biological replicates and 3 experimental units per diet group, so we sampled microbial populations of midguts and hindguts of 36 specimens.

### Bacterial community diversity

Community richness and diversity varied among gut segments and different diets (Table 1). All four richness and diversity indices were significantly higher in the midgut of both diet groups: M-FF (midgut-formula feed) > H-FF (hindgut-FF) (T_chao1_ = 4.954, *P *< 0.01; T_ACE_ = 4.850, *P *< 0.01; T_shannon_ = 4.938, *P *< 0.01; T_simpson_ = 2.326, *P *< 0.05), and M-SG (midgut-Sudan grass) > H-SG (T_chao1_ = 3.393, *P *< 0.01; T_ACE_ = 3.370, *P *< 0.01; T_shannon_ = 5.379, *P *< 0.01; T_simpson_ = 5.136, *P *< 0.01).When considering each diet independently, community richness of the FF group was significantly higher than that of the SG group (T_chao1_ = 3.408, *P *< 0.01; T_ACE_ = 3.582, *P *< 0.01). Nevertheless, community diversity was not significantly different between FF and SG groups (T_shannon_ = 1.908, P = 0.06; T_simpson_ = 0.841, p = 0.403). The highest community richness and diversity indices were found in the M-FF group (Chao1 = 1351.30 ± 345.69, ACE = 1413.41 ± 338.80, Shannon = 6.66 ± 2.03, and Simpson = 0.91 ± 0.16), while the lowest were found in the H-SG group (Chao1 = 527.37 ± 413.70, ACE = 533.72 ± 452.78, Shannon = 3.45 ± 1.38, and Simpson = 0.76 ± 0.09).

**Table 1:** Summary of alpha diversity estimators for microbial communities of four groups

Group	Richness estimates		Diversity estimates		Good’s coverage Mean ± SD
	Chao 1 Mean ± SD	ACE Mean ± SD	Shannon Mean ± SD	Simpson Mean ± SD	
M-FF	1351.30 ± 345.69	1413.41 ± 338.80	6.66 ± 2.03	0.91 ± 0.16	0.96 ± 0.01
M-SG	938.10 ± 413.70	975.91 ± 452.78	5.58 ± 1.38	0.91 ± 0.09	0.94 ± 0.05
H-FF	796.19 ± 345.69	850.99 ± 338.80	4.03 ± 2.03	0.81 ± 0.16	0.98 ± 0.01
H-SG	527.37 ± 413.70	533.72 ± 452.78	3.45 ± 1.38	0.76 ± 0.09	0.99 ± 0.05

M-FF, midgut samples of the group fed on formula fed; M-SG, midgut samples of the group fed on Sudan grass; H-FF, hindgut samples of the group fed on formula fed; H-SG, hindgut samples of the group fed on Sudan grass

### Bacterial community composition

**Fig. 1:**
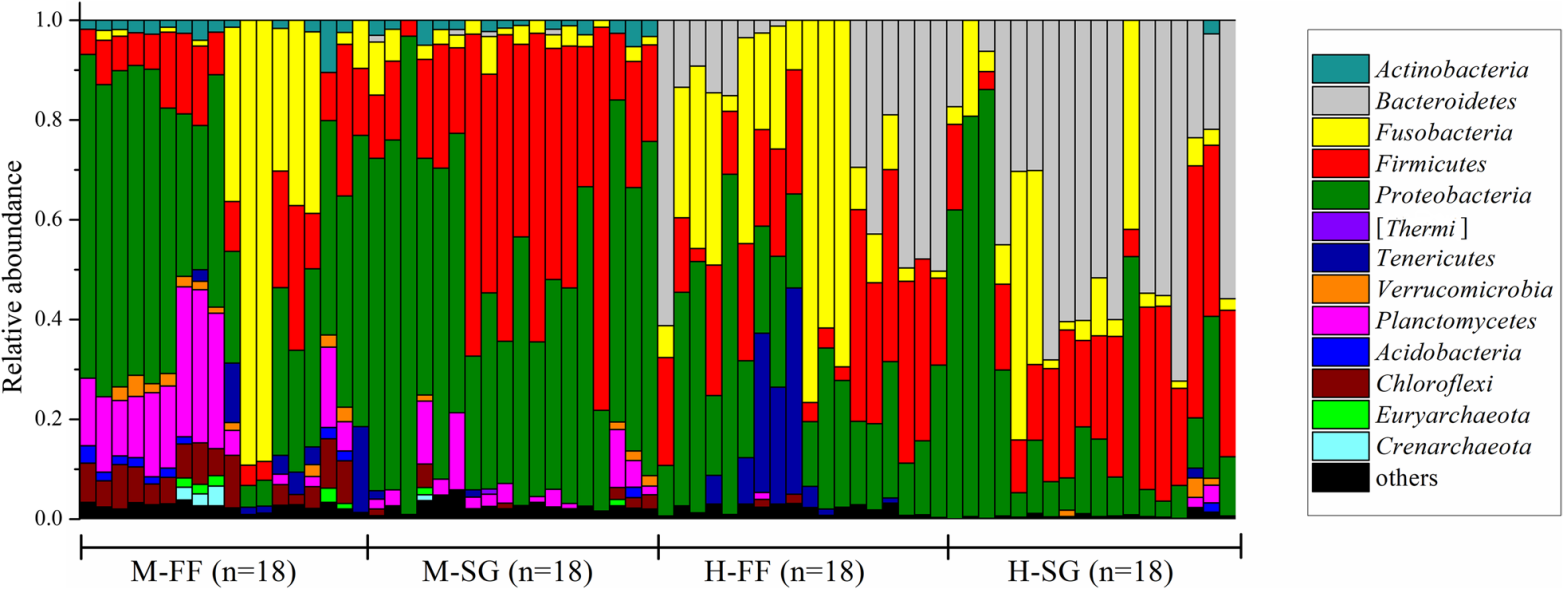
Composition of bacteria in four groups at the phylum level. Each bar represents the community of a sample. Only those phyla with mean relative abundance > 1% are shown; whereas low abundance phyla were assigned to ‘others’

Using the diet + segment grouping, at the phylum level, *Proteobacteria* (46.63 ± 19.7%), *Firmicutes* (23.52 ± 19.47%), *Fusobacteria* (11.02 ± 21.77%), *Planctomycetes* (7.70 ± 8.70%), and *Chloroflexi* (3.28 ± 3.34%) were dominant in the two midgut groups of samples (M-FF and M-SG; Fig. 1). However, *Bacteroidetes* (29.79 ± 24.22%), *Proteobacteria* (25.38% ± 21.40%), *Firmicutes* (21.52 ± 12.76%), *Fusobacteria* (18.15% ± 21.29%) and *Tenericutes* (3.53 ± 9.23%) were dominant in the two hindgut groups of samples (H-FF and H-SG; Fig. 1). At the intestinal segment level, *Bacteroidetes* were significantly more abundant in the H group (T = − 7.246, *P *< 0.001), while *Proteobacteria* were more abundant in the M group (T = 4.383, *P *< 0.001). At the diet level, the dominant phyla in the FF group were *Proteobacteria* (33.56 ± 19.02%), *Fusobacteria* (21.69 ± 26.49%), *Firmicutes* (16.74 ± 11.29%), *Bacteroidetes* (10.23 ± 17.77%), *Planctomycetes* (5.89 ± 9.12%), and *Tenericutes* (4.83 ± 9.38%), and dominant phyla in the SG group were *Proteobacteria* (38.46 ± 26.54%), *Firmicutes* (28.30 ± 18.67%), *Bacteroidetes* (20.10 ± 25.71%), *Fusobacteria* (7.48 ± 12.15%), *Planctomycetes* (2.17 ± 3.71%), and *Actinobacteria* (1.12 ± 1.45%). Statistical analysis indicated that *Fusobacteria* were significantly more abundant in the FF group than in the SG group (T = 2.927, *P *< 0.001). *Bacteroidetes* were more abundant in SG group than in FF group, but the difference was slightly above the selected statistical significance threshold (P = 0.063).

At the genus-level, the top ten most abundant genera differed among the four main sample groups (M and H, FF and SG; Additional file 1: Table S1). On average, *Bacteroides* species were more abundant (P = 0.076) in SG group (17.38 ± 22.55%) than in FF group (9.05 ± 16.17%). *Cetobacterium* were significantly higher (T = 2.672, *P *< 0.05) in FF group (18.53 ± 25.83%) than in SG group (5.89 ± 11.75%).

**Fig. 2:**
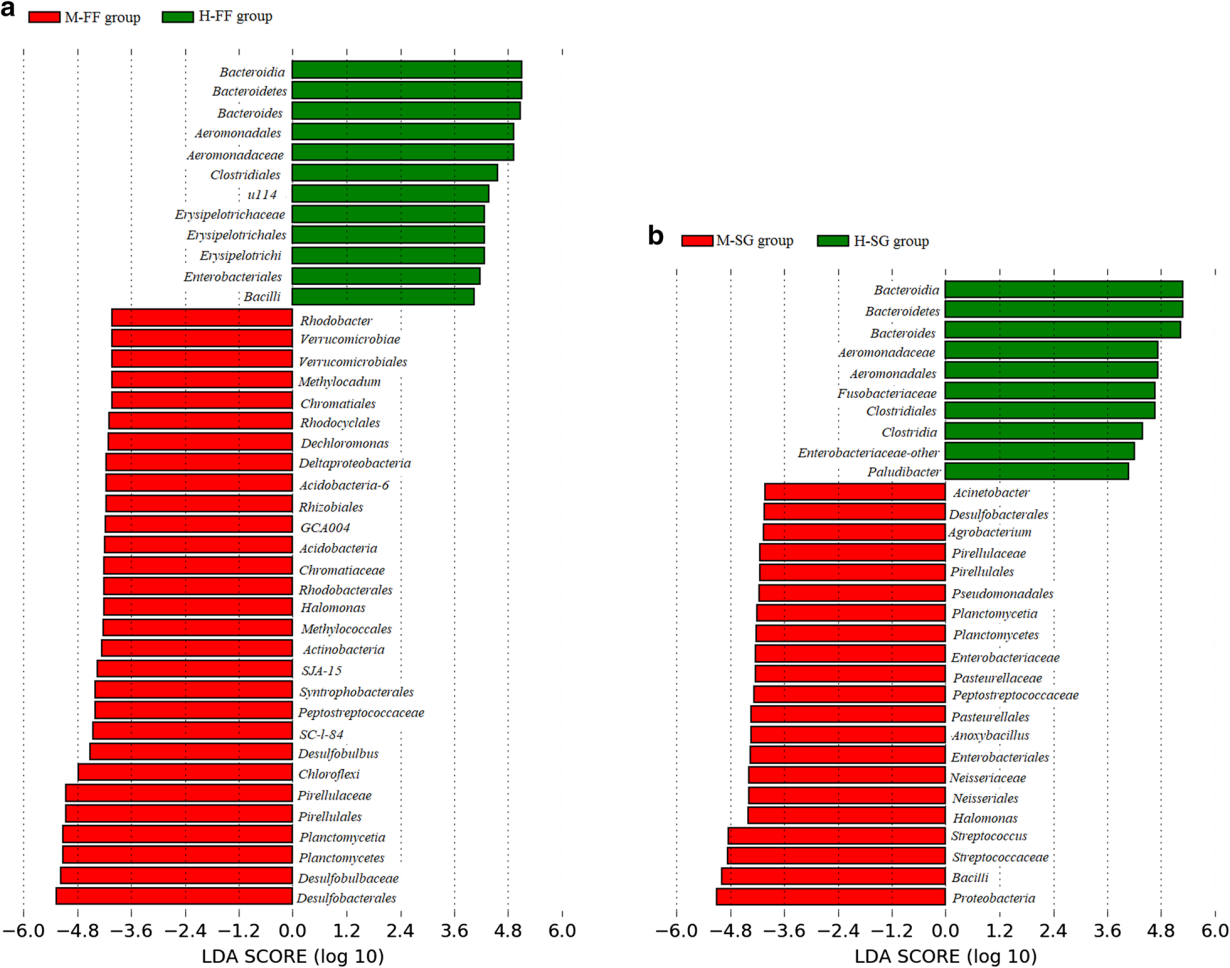
Bacterial taxa significantly different between the M-FF and H-FF groups (**a**) or between the M-SG and H-SG groups (**b**) identified by linear discriminant analysis coupled with effect size (LefSe) with LDA value set at 4.0

More than 700 bacterial taxa (genus or higher taxonomic level) significantly different (in terms of abundance) between the M-FF/H-FF and M-SG/H-SG group pairs were identified using Lefse with the LDA score value threshold set at 2.0. In the FF group, *Bacteroidetes* (mostly *Bacteroidia* and *Bacteroides*), *Erysipelotrichia* and *Aeromonadales* (mostly *Aeromonadaceae*) were the most enriched taxa in the hindgut, whereas *Desulfobacterium*, *Planctomycetes*, and *Pirelluales* (mostly *Pirelluaceae*) were the most significantly enriched taxa in the midgut (Fig. 2a). In the SG group, *Bacteroidetes* (mostly *Bacteroidia* and *Bacteroides*) and *Aeromonadaceae* were also the most enriched taxa in the hindgut, followed by *Fusobacteriaceae*, but *Proteobacteria*, *Bacilli* and *Streptococcaceae* (mostly *Streptococcus*) were the most significantly enriched taxa in the midgut (Fig. 2b).

### Relationships between bacterial communities of different gut segments and diets

A heatmap analysis at the family level showed that samples from the M group formed a single cluster, clearly distinct from the H group samples. PerMANOVA analysis revealed a significant difference (F = 51.29, P = 0.0001) in the composition of bacterial communities between M and H groups, but not between FF and SG groups (F = 1.316, P = 0.247). PerMANOVA with ‘adonis’ algorithm indicated that grass carp gut segment contributed 19.8% (*P *< 0.001) of the variation of gut bacterial communities, whereas diet contributed only 8.0% (*P *< 0.001) (Table 2). Similarly, VPA analysis indicated that gut segments explain 28% (*P *< 0.001) of the variation, and diet 14% (*P *< 0.001).

**Table 2:** Quantitative effects of gut segment and diet on the intestinal bacterial community assessed using permutational multivariate analyses of variance with Adonis function

	Gut segment		Diet		Gut segment: Diet	
	R^2^	P	R^2^	P	R^2^	P
Community variation	0.198	< 0.001	0.080	< 0.001	0.041	0.001

R^2^ values represent the proportion of the community variation explained by each variable

**Fig. 3:**
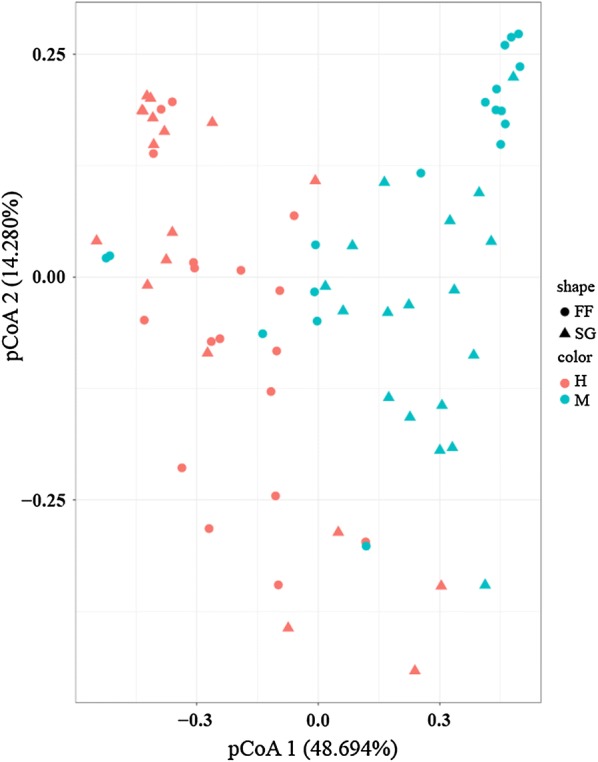
Principal coordinate analysis (PCoA) based on weighted UniFrac distances illustrating community dissimilarities over different gut segments and diet samples

PCoA results indicated that midgut and hindgut had significantly different bacterial compositions regardless of diet (P = 0.0001 in all cases, PerMANOVA based on weighted Unifrac; Fig. 3). After controlling for the gut compartment, we found a significant difference in bacterial composition between M-FF and M-SG samples (P = 0.0324; Additional file 1: Fig. S1), but not between H-FF and H-SG samples (P = 0.2688; Additional file 1: Fig. S2). We also determined the OTUs shared between these four groups of samples: M-FF and H-FF samples shared 1608 OTUs, M-SG and H-SG shared 1052, M-FF and M-SG shared 2401, and H-FF and H-SG groups shared 1272 OTUs (Additional file 1: Fig. S3).

### Functional prediction of the midgut and hindgut microbiota

**Fig. 4:**
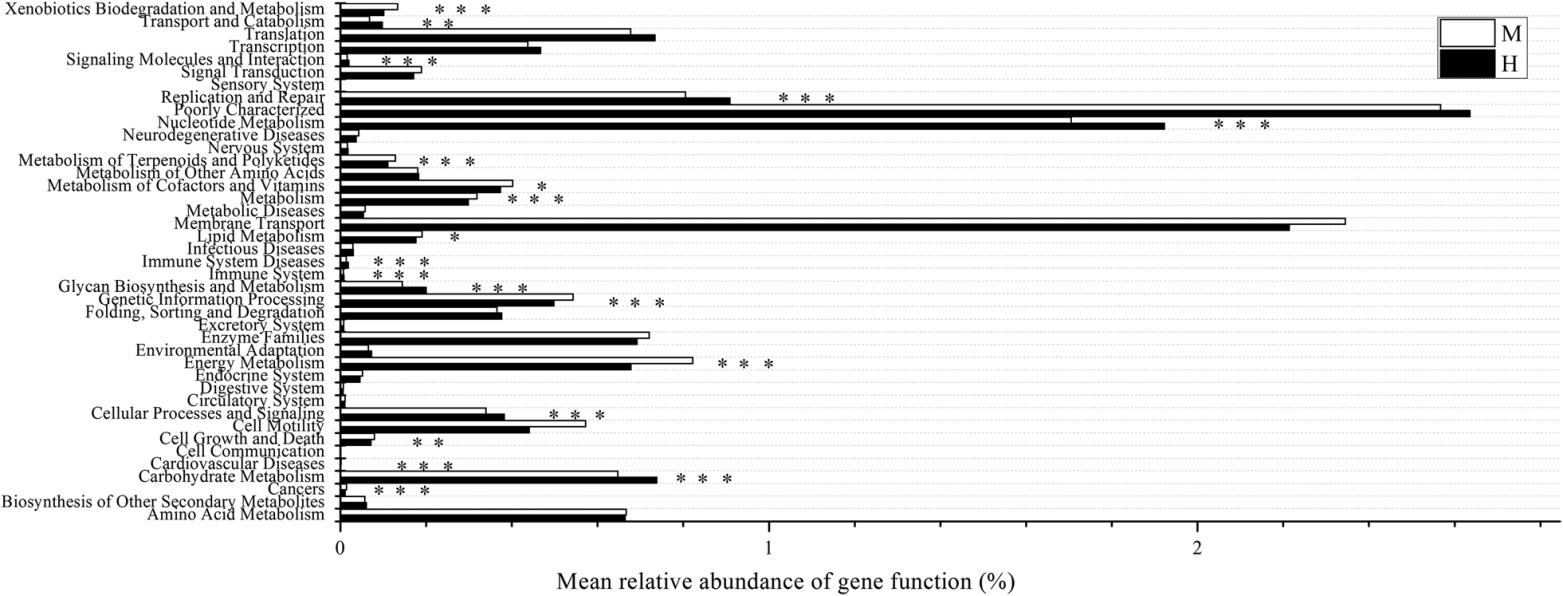
Functional profiling of midgut and hindgut microbial communities predicted by PICRUSt in the KEGG database (level 2). The significance level is indicated by *P < 0.05; **P < 0.01; ***P < 0.001

To infer the functional profiles of midgut and hindgut microbiota, microbial 16S rRNA sequence data were analyzed by PICRUST to predict the dominant gene families. KEGG database level 2 query assigned the genes to 41 functional groups, predominantly to ‘poorly characterized’, ‘membrane transport’, and ‘nucleotide metabolism’ (Fig. 4). Nineteen gene families exhibited significant (*P *< 0.05) differences between midgut and hindgut. The pathways these gene families were mainly associated with metabolic pathways: xenobiotics biodegradation and metabolism, nucleotide metabolism, metabolism of terpenoids and polyketides, metabolism of cofactors and vitamins, lipid metabolism, glycan biosynthesis and metabolism, energy metabolism, and carbohydrate metabolism. Some oxygen-independent pathways (especially fructose/mannose and starch/sucrose metabolisms) were also enriched in the hindgut samples (Additional file 1: Fig. S4).

## Discussion

Substantial research has been carried out in recent decades to better understand the complexity and diversity of gut microbiota in fish (Han et al. 2010; Sugita et al. 1985; Tran et al. 2017). Diet is known to be a very important factor influencing the intestinal bacterial composition. For example, in the Atlantic cod (*Gadusmorhua* L.), Gram-positive *Brochothrix* and *Carnobacterium* were dominant in the gut of a fish meal diet-fed fish, *Psychrobacter* dominated in the bioprocessed soy bean meal group, and *Carnobacterium*, *Chryseobacterium* and *Psychrobacter glacincola* dominated in the soy bean meal diet group (Ringø et al. 2006). However, the impact of different gut compartments on the bacterial composition remains unstudied in fish.

Our study provides a detailed comparison of bacterial communities in different gut segments in herbivorous fish, in combination with two strikingly different diets. Heatmap analysis indicated that midgut samples from both diet groups formed a single cluster, significantly different from the hindgut samples of both diet groups. This suggests that the composition of microbiota was impacted more substantially by the gut compartment than by the diet. However, large SD values observed in all of these analyses, as well as comparison with previous studies of this species (Hao et al. 2017b), indicate that individual variability also plays a major role in determining the microbial composition.

This dramatic difference in the microbiota composition between midgut and hindgut may be related to gut morphology and physicochemical conditions (Brune 1998; Brune and Friedrich 2000). Obligate anaerobes, including *Bacteroides* (*Bacteroidetes*), *Fusobacteriaceae* (*Fusobacteria*), and *Clostridiales* and *Erysipelotrichaceae* (*Firmicutes*), were significantly more abundant in hindgut samples than in midgut samples. *Proteobacteria*, however, were more abundant in the midgut samples. Predicted metagenomes also revealed increasing prevalence of anaerobic metabolism in hindgut in comparison to midgut, which included fructose and mannose metabolism, galactose metabolism, and starch and sucrose metabolism. The observed shift towards obligate anaerobes is expected, as the hindgut is characterized by extremely low oxygen concentrations in most animals (Mackie and White 2012). *Bacteroides* was also reported as the most abundant taxon in the distal gut segments of a broad spectrum of animal species, from mammals (sheep rectum) (Zhang et al. 2018) to insects (*Pachnoda ephippiata*, distal gut) (Egert et al. 2003). However, dominant taxa varied among the proximal gut samples of these three species: *Streptococcus* in sheep jejunum (Zhang et al. 2018), aerobic *Actinobacteria* in the midgut of *P. ephippiata* (Egert et al. 2003), and *Proteobacteria* in grass carp. Therefore, oxygen levels are the most likely explanation for the observed significant difference in the bacterial composition between bacterial communities of midgut but not hindgut samples of the two diet groups: in an aerobic environment, diet is the major factor determining the microbial composition, but as the environment turns anaerobic, it becomes hospitable only for a limited number of microbial taxa, resulting in shrinking microbial richness and diversity indices.

As diet is believed to be the most important force shaping the gut bacterial community (Ringø and Olsen 1999; Ringø et al. 2006; Tajima et al. 2001), we also studied the impacts of two very different diets: Sudan grass and formula feed. When each diet was considered independently, bacterial community richness of the FF group was significantly higher than that of the SG group. *Bacteroidetes* (non-significantly) and *Bacteroides* were more abundant in the SG group. The genome of *Bacteroides* is enriched in glycoside hydrolase and polysaccharide lyase genes, targeting the degradation of the plant cell wall polysaccharides (Hao et al. 2017b). Hence, high abundance of *Bacteroides* in the SG group probably reflects the high proportion of fiber in this diet. Similarly, gut microbiota of high-fiber diet consuming humans are highly enriched in *Bacteroidetes* (Maslowski and Mackay 2011). On the other hand, the *Cetobacterium* genus was significantly more abundant in the FF group (compared to SG group). This genus is known to be in a positive correlation with the production of acetic and propionic acids through peptone and glucose fermentation (Tsuchiya et al. 2008), and numerous gene families associated with protein digestion (peptidases) are present in the genome of *C. somerae*, which is an indigenous bacterium in the digestive tract of freshwater fish (Hao et al. 2017b). This could be an explanation behind the high abundance of this microbe in high-protein formula feed diet-fed fish (Hao et al. 2017a, b).

In conclusion, composition of the intestinal bacterial community is determined by a large number of factors, including the host’s diet, gut compartment, life history, genetics, and environmental factors (Ley et al. 2008; Wu et al. 2012), but diet is believed to outweigh the host’s genotype in shaping the gut microbiota (Carmody et al. 2015). We found that the opposite is true for gut segments: both PerMANOVA and VPA analyses indicated that gut segments explain a higher proportion of the variation in intestinal microbiota than the diet. Despite the large individual variability observed, these results indicate that we can reject our working hypothesis, as intestinal anatomy and physiology appear to be a stronger determinant in shaping the intestinal microbiota than host’s diet. Apart from the understanding of bacterial functions in different gut segments, this finding also bears relevance for the interpretation of past studies and design of future studies of intestinal microbiota, which should pay close attention to the intestinal segment variability.

## Additional file

**Additional file 1: Table S1.** The top 10 genera in four groups. **Figure S1.** Principal coordinate analysis (PCoA) based on weighted UniFrac distance illustrating community dissimilarities over M-FF group and M-SG groups. **Figure S2.** Principal coordinate analysis (PCoA) based on weighted UniFrac distance illustrating community dissimilarities over H-FF group and H-SG groups. **Figure S3.** Venn diagram showing the numbers of shared OTUs among different groups. **Figure S4.** Heat map showing five oxygen-independent pathways across midgut and hindgut predicted by PICRUSt.

## References

[CR1] Bäckhed F, Ding H, Wang T, Hooper LV, Koh GY, Nagy A, Semenkovich CF, Gordon JI (2004). The gut microbiota as an environmental factor that regulates fat storage. Proc Natl Acad Sci USA.

[CR2] Baker GC, Smith JJ, Cowan DA (2003). Review andre-analysis of domain-specific 16S primers. J Microbiol Methods.

[CR3] Benson AK, Kelly SA, Legge R, Ma F, Low SJ, Kim J, Zhang M, Oh PL, Nehrenberg D, Hua K, Kachman SD, Moriyama EN, Walter J, Peterson DA, Pomp D (2010). Individuality in gut microbiota composition is a complex polygenic trait shaped by multiple environmental and host genetic factors. Proc Natl Acad Sci USA.

[CR4] Brune A (1998). Termite guts: the world’s smallest bioreactors. Trends Biotechnol.

[CR5] Brune A, Friedrich M (2000). Microecology of the termite gut: structure and function on a microscale. Curr Opin Microbiol.

[CR6] Caporaso JG, Kuczynski J, Stombaugh J, Bittinger K, Bushman FD, Costello EK, Huttley GA (2010). QIIME allows analysis of high-throughput community sequencing data. Nat Methods.

[CR7] Carmody RN, Gerber GK, Luevano JM, Gatti DM, Somes L, Svenson KL, Turnbaugh PJ (2015). Diet dominates host genotype in shaping the murine gut microbiota. Cell Host Microbe.

[CR8] Chow S, Ruskey F (2003). Drawing area-proportional Venn and Euler diagrams. Lect Notes Comput Sci.

[CR9] Edgar RC, Haas BJ, Clemente JC, Quince C, Knight R (2011). UCHIME improves sensitivity and speed of chimera detection. Bioinformatics.

[CR10] Egert M, Wagner B, Lemke T, Brune A, Friedrich MW (2003). Microbial community structure in midgut and hindgut of the humus-feeding larva of *Pachnoda ephippiata* (Coleoptera: Scarabaeidae). Appl Environ Microbiol.

[CR11] Faith JJ, Guruge JL, Charbonneau M, Subramanian S, Seedorf H, Goodman AL, Clemente JC, Knight R, Heath AC, Leibel RL, Rosenbaum M, Gordon JI (2013). The long-term stability of the human gut microbiota. Science.

[CR12] Feng L, Xia JH, Bai ZY, Fu JJ, Li JL, Yue GH (2009). High genetic diversity and substantial population differentiation in grass carp (*Ctenopharyngodon idella*) revealed by microsatellite analysis. Aquaculture.

[CR13] Gacias M, Gaspari S, Santos PM, Tamburini S, Andrade M, Zhang F, Shen N, Tolstikov V, Kiebish MA, Dupree JL, Zachariou V, Clemente JC, Casaccia P (2016). Microbiota-driven transcriptional changes in prefrontal cortex override genetic differences in social behavior. Elife.

[CR14] Gilbert SF, Sapp J, Tauber AI (2012). A symbiotic view of life: we have never been individuals. Q Rev Biol.

[CR15] Hammer-Muntz O, Harper DAT, Ryan PD (2001). PAST: Paleontological Statistics Software Package for education and data analysis. Palaeontol Electron.

[CR16] Han S, Liu Y, Zhou Z, He S, Cao Y, Shi P, Yao B, Ringø E (2010). Analysis of bacterial diversity in the intestine of grass carp (*Ctenopharyngodon idellus*) based on 16S rDNA gene sequences. Aquac Res.

[CR17] Hao YT, Wu SG, Jakovlić I, Zou H, Li WX, Wang GT (2017). Impacts of diet on hindgut microbiota and short-chain fatty acids in grass carp (*Ctenopharyngodon idellus*). Aquac Res.

[CR18] Hao YT, Wu SG, Xiong F, Tran NT, Jakovlić I, Zou H, Li WX, Wang GT (2017). Succession and fermentation products of grass carp (*Ctenopharyngodon idellus*) hindgut microbiota in response to an extreme dietary shift. Front Microbiol.

[CR19] Langille MG, Zaneveld J, Caporaso JG, McDonald D, Knights D, Reyes JA, Clemente JC, Burkepile DE, Vega Thurber RL, Knight R, Beiko RG, Huttenhower C (2013). Predictive functional profiling of microbial communities using 16S rRNA marker gene sequences. Nat Biotechnol.

[CR20] Ley RE, Peterson DA, Gordon JI (2006). Ecological and evolutionary forces shaping microbial diversity in the human intestine. Cell.

[CR21] Ley RE, Lozupone CA, Hamady M, Knight R, Gordon JI (2008). Worlds within worlds: evolution of the vertebrate gut microbiota. Nat Rev Microbiol.

[CR23] Li H, Li T, Tu B, Kou Y, Li X (2017). Host species shapes the co-occurrence patterns rather than diversity of stomach bacterial communities in pikas. Appl Microbiol Biotechnol.

[CR24] Lozupone C, Lladser ME, Knights D, Stombaugh J, Knight R (2011). UniFrac: an effective distance metric for microbial community comparison. ISME J.

[CR25] Mackie RI, White BA (2012). Gastrointestinal microbiology: Volume 1 gastrointestinal ecosystems and fermentations.

[CR26] Magoč T, Salzberg SL (2011). FLASH: fast length adjustment of short reads to improve genome assemblies. Bioinformatics.

[CR27] Maslowski KM, Mackay CR (2011). Diet, gut microbiota and immune responses. Nat Immunol.

[CR28] Miyake S, Ngugi DK, Stingl U (2015). Diet strongly influences the gut microbiota of surgeonfishes. Mol Ecol.

[CR29] Mowat AM, Agace WW (2014). Regional specialization within the intestinal immune system. Nat Rev Immunol.

[CR30] Ni DS, Wang JG (1999). Biology and diseases of grass carp.

[CR31] Ni J, Yan Q, Yu Y, Wu H, Chen F (2017). Dispersal patterns of endogenous bacteria among grass carp (*Ctenopharyngodon idellus*) guts. Iran J Fish Sci.

[CR32] Parks DH, Tyson GW, Hugenholtz P, Beiko RG (2014). STAMP: statistical analysis of taxonomic and functional profiles. Bioinformatics.

[CR33] Perea K, Perz K, Olivo SK, Williams A, Lachman M, Ishaq SL, Thomson J, Yeoman CJ (2017). Feed efficiency phenotypes in lambs involve changes in ruminal, colonic, and small-intestine-located microbiota. J Anim Sci.

[CR34] Qin J, Li R, Raes J, Arumugam M, Burgdorf KS, Manichanh C, Nielsen T, Pons N, Levenez F, Yamada T, Mende DR, Li J, Xu J, Li S, Li D, Cao J, Wang B, Liang H, Zheng H, Xie Y, Tap J, Lepage P, Bertalan M, Batto JM, Hansen T, Le Paslier D, Linneberg A, Nielsen HB, Pelletier E, Renault P, Sicheritz-Ponten T, Turner K, Zhu H, Yu C, Li S, Jian M, Zhou Y, Li Y, Zhang X, Li S, Qin N, Yang H, Wang J, Brunak S, Doré J, Guarner F, Kristiansen K, Pedersen O, Parkhill J, Weissenbach J, Bork P, Ehrlich SD, Wang J, MetaHIT Consortium (2010). A human gut microbial gene catalogue established by metagenomic sequencing. Nature.

[CR35] Rawls JF, Mahowald MA, Ley RE, Gordon JI (2006). Reciprocal gut microbiota transplants from zebrafish and mice to germ-free recipients reveal host habitat selection. Cell.

[CR36] Ringø E, Olsen R (1999). The effect of diet on aerobic bacterial flora associated with intestine of Arctic charr (*Salvelinus alpinus* L.). J Appl Microbiol.

[CR37] Ringø E, Sperstad S, Myklebust R, Refstie S, Krogdahl A (2006). Characterisation of the microbiota associated with intestine of Atlantic cod (*Gadus morhua* L.): the effect of fish meal, standard soybean meal and a bioprocessed soybean meal. Aquaculture.

[CR38] Segata N, Izard J, Waldron L, Gevers D, Miropolsky L, Garrett WS, Huttenhower C (2011). Metagenomic biomarker discovery and explanation. Genome Biol.

[CR39] Sire MF, Vernier JM (1992). Intestinal absorption of protein in teleost fish. Comp Biochem Physiol A Physiol.

[CR40] Stevenson KJ (2015). Review of OriginPro 8.5. J Am Chem Soc.

[CR41] Sugita H, Tokuyama K, Deguchi Y (1985). The intestinal microflora of carp *Cyprinus carpio*, grass carp *Ctenopharyngodon idella* and tilapia *Sarotherodon niloticus*. Bull Jpn Soc Sci Fish.

[CR42] Tajima K, Aminov R, Nagamine T, Matsui H, Nakamura M, Benno Y (2001). Diet-dependent shifts in the bacterial population of the rumen revealed with real-time PCR. Appl Environ Microbiol.

[CR43] Torok VA, Ophel-Keller K, Loo M, Hughes RJ (2008). Application of methods for identifying broiler chicken gut bacterial species linked with increased energy metabolism. Appl Environ Microbiol.

[CR44] Tran NT, Xiong F, Hao YT, Zhang J, Wu SG, Wang GT (2017). Two biomass preparation methods provide insights into studying microbial communities of intestinal mucosa in grass carp (*Ctenopharyngodon idellus*). Aquacult Res.

[CR45] Tsuchiya C, Sakata T, Sugita H (2008). Novel ecological niche of *Cetobacterium somerae*, an anaerobic bacterium in the intestinal tracts of freshwater fish. Lett Appl Microbiol.

[CR46] Wu GD, Chen J, Hoffmann C, Bittinger K, Chen Y-Y, Keilbaugh SA, Bewtra M, Knights D, Walters WA, Knight R (2011). Linking long-term dietary patterns with gut microbial enterotypes. Science.

[CR48] Wu S, Wang G, Angert ER, Wang W, Li W, Zou H (2012). Composition, diversity, and origin of the bacterial community in grass carp intestine. PLoS ONE.

[CR50] Wu S, Ren Y, Peng C, Hao Y, Xiong F, Wang G, Li W, Zou H, Angert ER (2015). Metatranscriptomic discovery of plant biomass-degrading capacity from grass carp intestinal microbiomes. FEMS Microbiol Ecol.

[CR51] Xiong J, Zhu J, Dai W, Dong C, Qiu Q, Li C (2017). Integrating gut microbiota immaturity and disease-discriminatory taxa to diagnose the initiation and severity of shrimp disease. Environ Microbiol.

[CR52] Ye L, Amberg J, Chapman D, Gaikowski M, Liu W (2014). Fish gut microbiota analysis differentiates physiology and behavior of invasive Asian carp and indigenous American fish. ISME J.

[CR53] Zhang C, Zhang M, Pang X, Zhao Y, Wang L, Zhao L (2012). Structural resilience of the gut microbiota in adult mice under high-fat dietary perturbations. ISME J.

[CR54] Zhang J, Xiong F, Wang GT, Li WX, Li M, Zou H, Wu SG (2017). The influence of diet on the grass carp intestinal microbiota and bile acids. Aquacult Res.

[CR55] Zhang H, Shao M, Huang H, Wang S, Ma L, Wang H, Hu L, Wei K, Zhu R (2018). The dynamic distribution of small-tail Han sheep microbiota across different intestinal segments. Front Microbiol.

