# Supplementary material for: Gut segments outweigh the diet in shaping the intestinal microbiota composition in grass carp Ctenopharyngodon idellus
Source: AMB Express. 2019 Apr 6;9:44. doi: 10.1186/s13568-019-0770-0 (PMC6451743; doi:10.1186/s13568-019-0770-0)
Supplement: Supplementary file 1 — Additional file 1: Table S1. The top 10 genera in four groups. Figure S1. Principal coordinate analysis (PCoA) based on weighted UniFrac distance illustrating community dissimilarities over M-FF group and M-SG groups. Figure S2. Principal coordinate analysis (PCoA) based on weighted UniFrac distance illustrating community dissimilarities over H-FF group and H-SG groups. Figure S3. Venn diagram showing the numbers of shared OTUs among different groups. Figure S4. Heat map showing five oxygen-independent pathways across midgut and hindgut predicted by PICRUSt. [file 13568_2019_770_MOESM1_ESM.pdf]

AMB Express

Gut segments outweigh the diet in shaping the intestinal microbiome composition in grass carp *Ctenopharyngodon idellus*

Wenwen Feng[1,2] . Jing Zhang[1,2] . IvanJakovlić[3] . Fan Xiong[1,2] . Shangong Wu[1,2] . Hong Zou[1,2] . Wenxiang Li[1,2] . MingLi[1,2] . Guitang Wang[1,2]

[1]*Key Laboratory of Aquaculture Disease Control, Ministry of Agriculture, and State Key Laboratory of Freshwater Ecology and Biotechnology, Institute of Hydrobiology, Chinese Academy of Sciences, Wuhan 430072, China*

[2]*University of Chinese Academy of Sciences, Beijing 100049, China*

[3]*Bio-Transduction Lab, Wuhan, 430072, China*

Shangong Wu, Fax: 86-027-68780123, Telephone number: 027-68780655

E-mail: wusgz@ihb.ac.cn.

Table S1. The top 10 genera in four groups

Other 1: *Enterobacteriaceae*; other 2: *Lachnospiraceae*

M group, all midgut samples; H group, all hindgut samples; FF group, all samples fed on formula fed; SG group, all samples fed on Sudan grass

Top 10 genera	M group	H group	FF group	SG group
1	*Cetobacterium*	*Bacteroides*	*Cetobacterium*	*Bacteroides*
2	*Streptococcus*	*Cetobacterium*	*Bacteroides*	*Streptococcus*
3	*Halomonas*	Other 2	u114	*Cetobacterium*
4	Other 1	u114	*Paenibacillus*	Other 1
5	*Anoxybacillus*	*Paludibacter*	*Desulfobulbus*	*Halomonas*
6	*Desulfobulbus*	*Paenibacillus*	*Halomonas*	*Anoxybacillus*
7	*Shewanella*	*Fusobacterium*	*Dechloromonas*	*Paludibacter*
8	*Acinetobacter*	*Leptotrichia*	Other 1	*Shewanella*
9	*Agrobacterium*	*Bacillus*	*Bacillus*	*Acinetobacter*
10	*Dechloromonas*	Other 1	*Methylocaldum*	*Leptotrichia*

Figure legends

Figure S1. Principal coordinate analysis (PCoA) based on weighted UniFrac distance illustrating community dissimilarities over M-FF group and M-SG groups.

Figure S2. Principal coordinate analysis (PCoA) based on weighted UniFrac distance illustrating community dissimilarities over H-FF group and H-SG groups.

Figure S3. Venn diagram showing the numbers of shared OTUs among different groups.

Figure S4. Heat map showing five oxygen-independent pathways across midgut and hindgut predicted by PICRUSt.

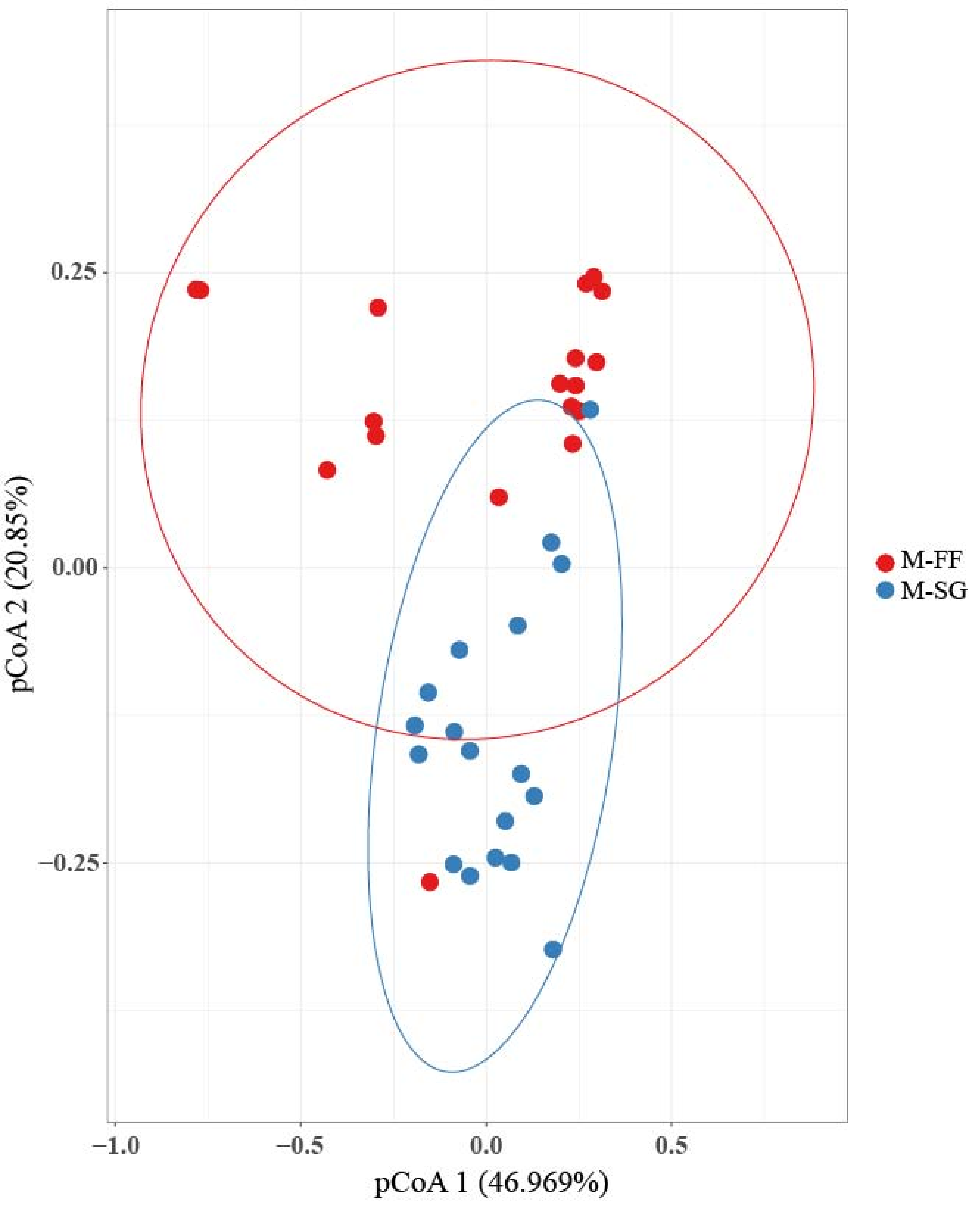

0.25
0.00
−0.25
−1.0
−0.5
0.0
0.5
pCoA 1 (46.969%)
pCoA 2 (20.85%)
M-FF
M-SG

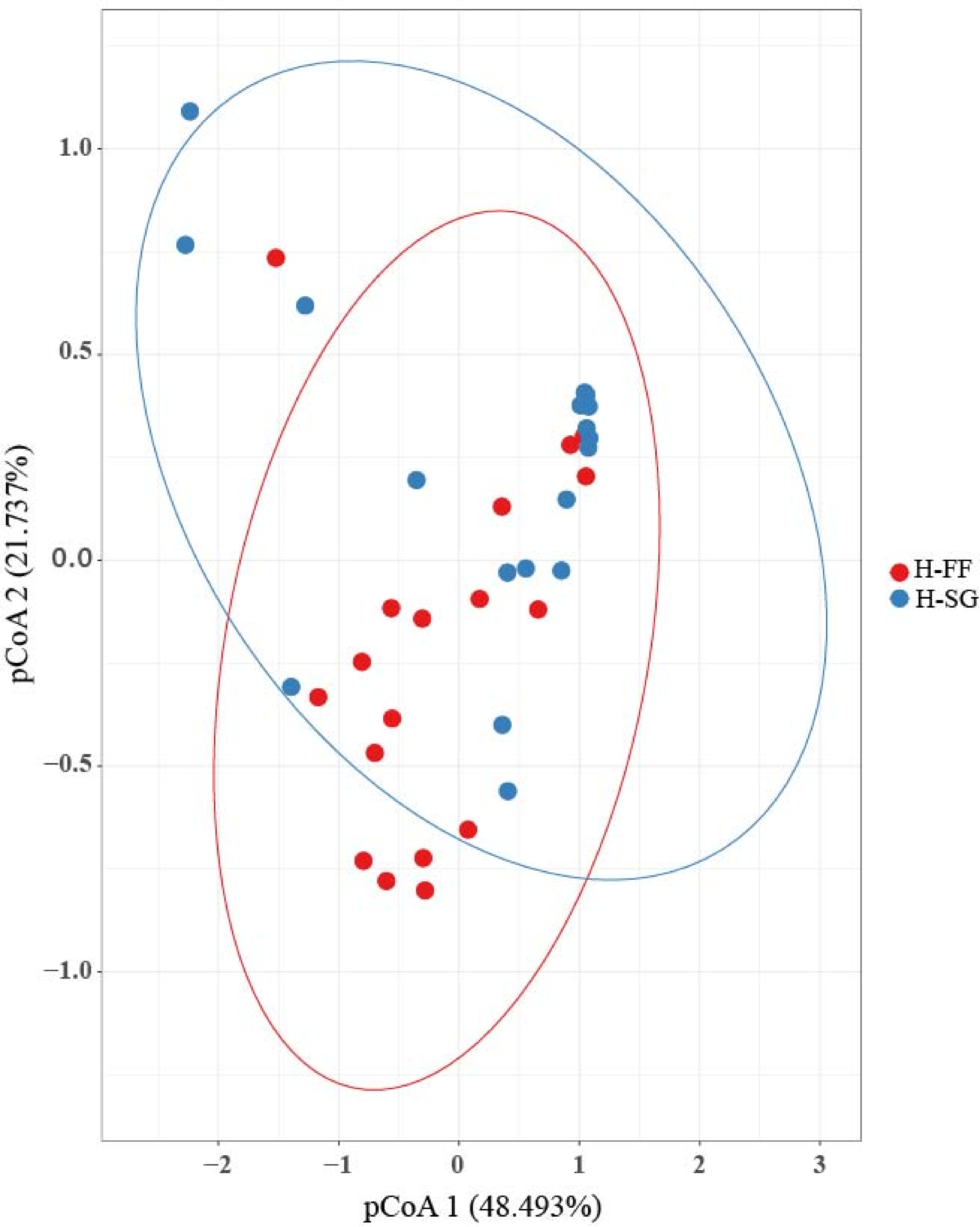

1.0
0.5
0.0
−0.5
−1.0
pCoA 2 (21.737%)
−2
−1
0
1
2
3
pCoA 1 (48.493%)
H-FF
H-SG

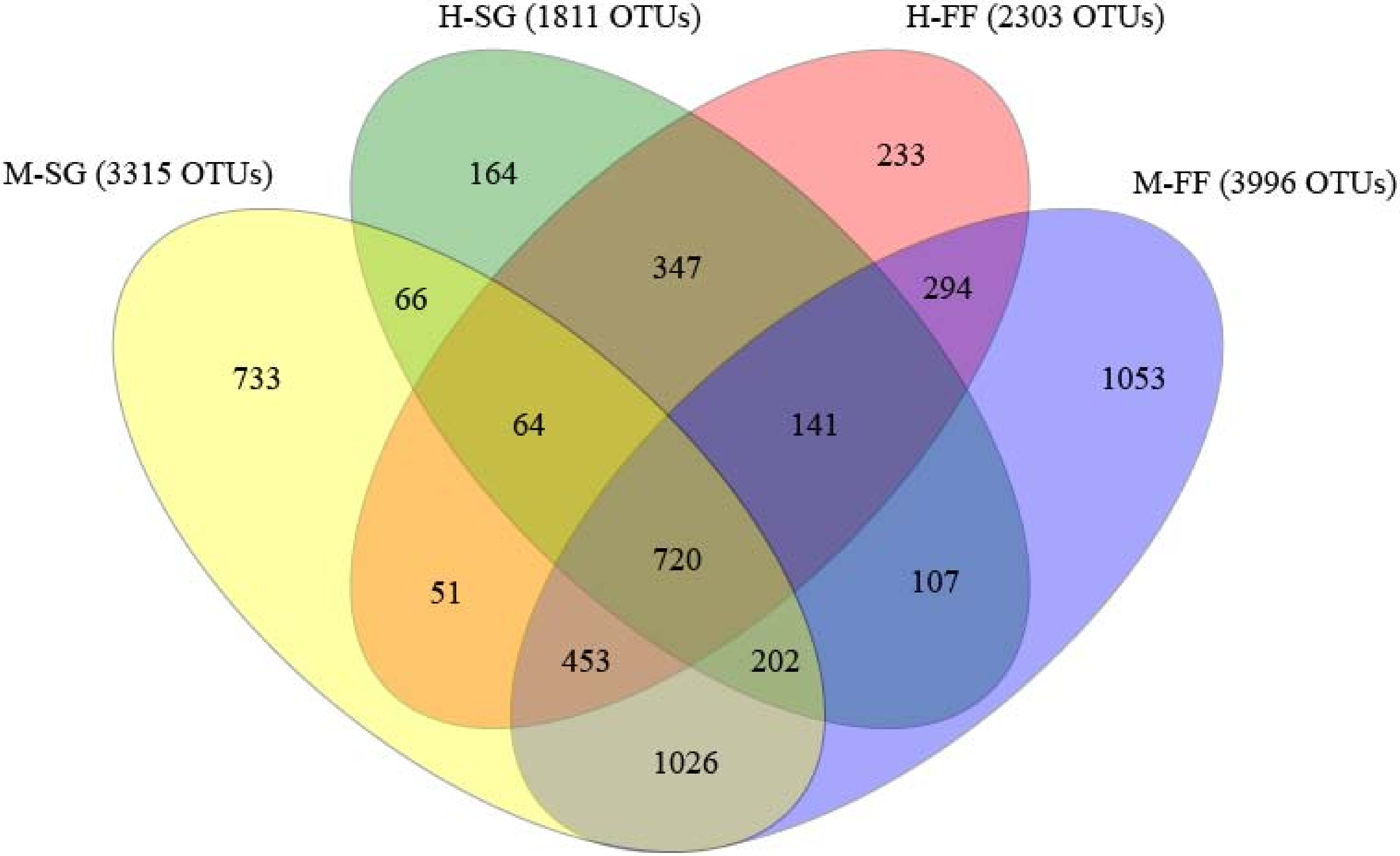
H-SG (1811 OTUs)
H-FF (2303 OTUs)
M-SG (3315 OTUs)
M-FF (3996 OTUs)
164
233
66
347
294
733
64
141
1053
720
51
107
453
202
1026

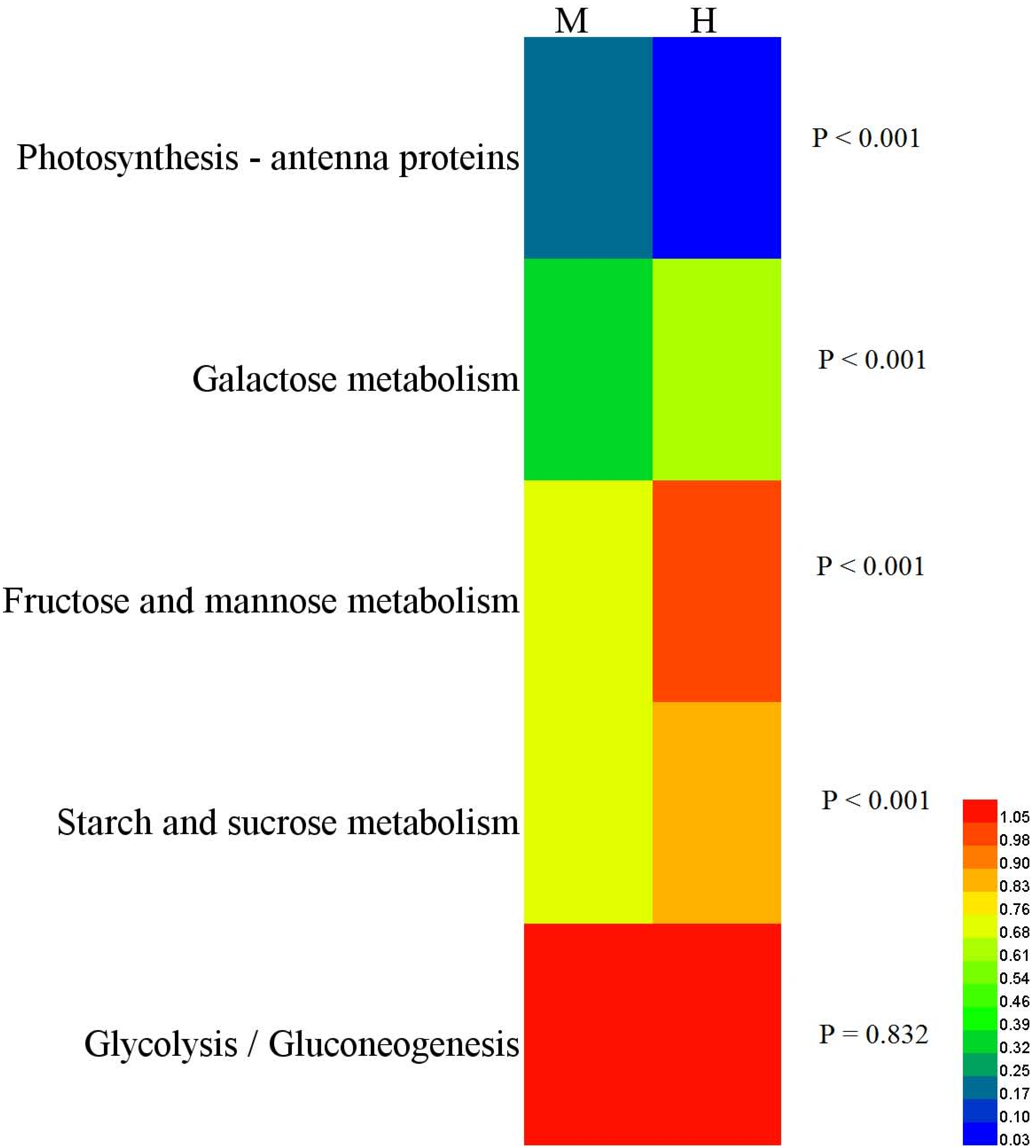
M
H
Photosynthesis - antenna proteins
P < 0.001
Galactose metabolism
P < 0.001
Fructose and mannose metabolism
P < 0.001
Starch and sucrose metabolism
P < 0.001
Glycolysis / Gluconeogenesis
P = 0.832
1.05
0.98
0.90
0.83
0.76
0.68
0.61
0.54
0.46
0.39
0.32
0.25
0.17
0.10
0.03
